# Modelling normal and impaired letter recognition: Implications for understanding pure alexic reading

**DOI:** 10.1016/j.neuropsychologia.2012.07.031

**Published:** 2012-10

**Authors:** Ya-Ning Chang, Steve Furber, Stephen Welbourne

**Affiliations:** a Neuroscience and Aphasia Research Unit (NARU), University of Manchester, Manchester M13 9PL, UK; b School of Computer Science, University of Manchester, Manchester M13 9PL, UK

**Keywords:** Letter recognition, Letter confusability, Pure alexia, Computational modelling

## Abstract

Letter recognition is the foundation of the human reading system. Despite this, it tends to receive little attention in computational modelling of single word reading. Here we present a model that can be trained to recognise letters in various spatial transformations. When presented with degraded stimuli the model makes letter confusion errors that correlate with human confusability data. Analyses of the internal representations of the model suggest that a small set of learned visual feature detectors support the recognition of both upper case and lower case letters in various fonts and transformations. We postulated that a damaged version of the model might be expected to act in a similar manner to patients suffering from pure alexia. Summed error score generated from the model was found to be a very good predictor of the reading times of pure alexic patients, outperforming simple word length, and accounting for 47% of the variance. These findings are consistent with a hypothesis suggesting that impaired visual processing is a key to understanding the strong word-length effects found in pure alexic patients.

## Highlights

► We develop a connectionist letter recognition model which provides the link between visual input and letter recognition. ► The model can deal with the invariance problem and generalise to previously unseen letters. ► The model can extract key features for letter recognition. ► We demonstrate the model can simulate confusability effects in normal readers and predict PA patients' RTs. ► Impaired visual processing is a key to understanding the strong word-length effects found in PA patients.

## 1 Introduction

During reading, the letters comprising a word are recognised, combined and then converted into meaning and sound. Letter recognition is the earliest reading specific process and provides essential information required for lexical-level processing. Despite this, models of single word reading in the literature do not generally include a mechanism for letter recognition. This is particularly true for models based on parallel distributed processing (Harm & Seidenberg, 2004; Plaut, McClelland, Seidenberg, & Patterson, 1996; Seidenberg & McClelland, 1989). Other reading models (Coltheart, Rastle, Perry, Langdon, & Ziegler, 2001; Perry, Ziegler, & Zorzi, 2007) have a processing stage of letter recognition which incorporates the interactive activation (IA) model (McClelland & Rumelhart, 1981). However, even in these cases the functions of their visual systems are limited by the fact that the IA model uses a predefined set of visual features as its input, rather than a purely visual representation of the letters. This means that important phenomena related to visual processes including letter confusability effects, font invariance, size invariance and feature extraction are beyond the scope of these models. In addition, there has been growing concern about the issue of how letter position information is encoded in words. This has inspired several new models such as the theoretical model based on open-bigrams coding (Grainger & Van Heuven, 2003), the SERIOL model (Whitney, 2001) and the SOLAR model (Davis, 2010). These models focus more closely on the level of orthographic processing, but they still do not contain a visual input stage and so cannot address the effects outlined above.

Human observers can recognise letters in various transformations quickly, efficiently and accurately; presumably by making use of an efficient representational system. Only under degraded viewing conditions does this break down revealing a characteristic pattern of letter confusability (Bouma, 1971; Gilmore, Hersh, Caramazza, & Griffin, 1979; Loomis, 1982; Townsend, 1971; Van der Heijden, Malhas, & Van den Roovaart, 1984). To perform the same task computationally is non-trivial, which may be why most existing models of reading avoid simulating this stage of processing.

To understand the nature of letter representations, two broad theories of visual recognition have been proposed. The template-matching account (Neisser, 1967) assumes that letter recognition is achieved by a process of matching the letter stimulus to an internal template. However, the obvious problem with this account, as noted by Høffding (1892) is that people are capable of recognising letters, irrespective of their size, orientation or font. It is very difficult to believe that we have stored templates for all possible transformations of a letter. To avoid this some normalisation process would be necessary to match to a limited number of internal templates in canonical forms. However, it is not clear whether this normalisation procedure is biologically plausible (Rolls & Deco, 2002). The alternative to the template theory is the feature-based approach (Gibson, 1969). By this account the visual features of letters are extracted in the early stage of the processing, and then those features are compared with lists of features stored in memory. The feature-based theory is supported by findings from many studies. Probably, the strongest evidence comes from a neurophysiological study by Hubel and Wiesel in 1959. They found that neurons in the primary visual cortex (V1) of cats showed a strong feature preference suggesting that feature detection is a key part of early visual processing. More recently, Pelli, Burns, Farell, and Moore-Page (2006) examined a wide range of viewing conditions with human observers varying in their ages and experience of identifying letters. They found that the efficiency for letter identification was inversely proportional to letter complexity, almost independent of other conditions (e.g., letter contrast, presentation duration, eccentricity and letter size). Fiset et al. (2008) examined what visual features contributed to human letter recognition by using a novel classification image technique, called bubbles. The basis of this technique is that the recognition performance should be impaired if the crucial information is masked; on the other hand, performance should be less affected if the information is not necessary. In their study, the visual information on five spatial frequency bands decomposed from letter images was masked and then partially revealed with random bubbles at different locations on the image. The number of the bubbles was controlled to maintain an accuracy rate close to 50% correct. The effective information required for letter recognition was identified by computing the correlations between the accuracy and the location of bubbles on the space dimension for each spatial frequency. Fiset et al. found that the most effective features for letter recognition were line terminations across upper and lower case letters in Arial font.

Another way to identify useful letter features is to measure the letter confusability between letters (Bouma, 1971; Gilmore et al., 1979; Loomis, 1982; Townsend, 1971; Van der Heijden et al., 1984). Letter confusability is assumed to reflect the visual similarity between letters that share the same features. The traditional approach to producing a letter confusability matrix is to ask subjects to identify letters in a degraded viewing condition, such as viewing degraded stimuli or viewing stimuli for a very brief exposure time. Alternatively, some researchers have attempted to obtain letter confusion values in a normal viewing condition (Courrieu, Farioli, & Grainger, 2004; Jacobs, Nazir, & Heller, 1989). For example, Courrieu et al. (2004) recorded the subjects' reaction times to discriminate two different letters. The inverse discrimination times were then used as a proxy for the confusion values between letters where a longer reaction time indicates two letters are more similar to each other.

### 1.1 Pure alexia

Pure alexia (PA) is a neuropsychological deficit generally caused by lesions in the left ventral occipito-temporal region (Damasio & Damasio, 1983). PA patients are defined by extremely slow reading which highly depends on word length and sometimes show a letter-by-letter (LBL) reading strategy; by contrast, normal readers only show small effects of word length in reading performance (Weekes, 1997). Pure alexia is of interest here because there is considerable evidence indicating that the deficit occurs in the early stage of visual processing, either for processes which are specific to letter encoding (Arguin, Fiset, & Bub, 2002; Fiset, Arguin, Bub, Humphreys, & Riddoch, 2005; Fiset, Gosselin, Blais, & Arguin, 2006), or for processes that subserve more generic visual operations required for processing both orthographic and non-orthographic stimuli (Behrmann, Nelson, & Sekuler, 1998; Farah & Wallace, 1991; Mycroft, Behrmann, & Kay, 2009). The strong length effect seen in PA patients is thought to indicate that each letter in a word is explicitly recognised by a serial processing mechanism so the reaction times are linearly dependent on word length (Warrington & Shallice, 1980). However, recent research by Arguin et al. (2002) proposes that PA patients can still process letters in parallel like normal readers (albeit that their visual processing is impaired) and the summed letter confusability is a primary factor that predicts the slow reading times of PA patients. This is supported by Fiset et al. (2005) who showed that the abnormally strong word-length effect was eliminated if the summed letter confusability of letter strings was matched across different word lengths. Fiset et al. (2005) further argued that, if PA patients were using a serial mechanism to process letters in a word sequentially instead of impaired parallel processing, and the rate of serial processing is linearly correlated with the letter confusability of the individual letters, then it might explain why matching the summed letter confusability of letter strings across different length groups could result in the abolition of the word-length effect. This would be correct only if PA patients were also sensitive to the letter confusion values of individual letters. However, they found the performance of PA patients was not affected by letter confusability when they were asked to identify individual letters either in isolation or in a word context. On the basis of their results, Fiset et al. (2005) suggest that parallel processing still occurs in PA patients although it is impaired. Only when the signal to noise ratio of the visual signal is too low would PA patients adopt a serial strategy such as attention shift.

Another interesting aspect of the PA reading performance is that PA patients are sensitive to several lexical variables such as word frequency (Behrmann, Plaut, & Nelson, 1998; Fiset, Arguin, & McCabe, 2006; Johnson & Rayner, 2007; Montant & Behrmann, 2001), orthographic neighbourhood size (Arguin et al., 2002; Fiset, Arguin et al., 2006; Montant & Behrmann, 2001), age-of-acquisition (Cushman & Johnson, 2011), concreteness (Behrmann, Plaut et al., 1998) and word imageability (Behrmann, Plaut et al., 1998; Behrmann, Shomstein, Black, & Barton, 2001; Fiset, Arguin et al., 2006; Roberts, Lambon Ralph, & Woollams, 2010). This sensitivity to these lexical variables might have an important implication for the understanding of the reading mechanism in PA patients. If the patients were using a completely different serial mechanism for transforming orthography to phonology why would the reaction times be affected by these variables? However, if the system is the same interactive parallel system as for the normal readers, but with impaired early visual processing then it makes sense that these top down variables would still affect reaction times.

### 1.2 Interactive activation model

As far as we are aware only a very few reading models include a stage of letter processing (Coltheart et al., 2001; Perry et al., 2007), and in these cases the recognition systems are all based on the interactive activation (IA) model (McClelland & Rumelhart, 1981; Rumelhart & McClelland, 1982). The IA model consists of three stages of processing: feature, letter and word levels. Word recognition starts from using a set of predefined visual features as input, moves on to the identification of constituent letters, and then finally to the activation of all candidate words. The communication between the word level and letter level is through bidirectional connections, and the communication between the feature level and letter level is through feedforward unidirectional connections. The IA model provides many valuable insights into the process of letter perception. For example, it captures the word superiority effect in which there is greater accuracy of letter detection in words than either in pseudowords or isolated letters showing that this effect is dependent on the feedback connections from the word level to letter level. It also shows a contextual facilitation of letter recognition. A recent study by Rey, Dufau, Massol, and Grainger (2009) examined different connectivity parameters within the IA model by using event-related potentials (ERPs) during letter identification. Their results supported a version of the IA model which had both feedforward and feedback connections between feature and letter levels, and lateral inhibition at the letter level. Despite its considerable success, the IA model does have some intrinsic limitations. Of particular relevance here is the fact that the IA model was not designed to learn to capture visual features. Instead the model was designed to recognise a particular set of predetermined features based on analyses by Rumelhart and Siple (1974). This means that effects of invariance (e.g., fonts, sizes and positions) are beyond the scope of the model. In addition it is unclear how the model would capture letter confusability effects, although Finkbeiner and Coltheart (2009) have proposed that adjusting the letter to letter inhibition in the model might allow for some modelling of confusability. However, this would still be unable to capture differences in confusability arising from differing font types. Obviously, all the reading models that include the IA model as the visual recognition component will unavoidably inherit these intrinsic limitations.

The present study is an attempt to show how a connectionist letter recognition model can provide the link between visual input and letter recognition. In doing so, we demonstrate how the model can (1) deal with the problem of size and shape invariance; (2) generalise to previously unseen letters; (3) extract key features for letter recognition; (4) simulate confusability effects in normal readers; (5) account for important behavioural data from PA patients.

## 2 Simulation 1

Simulation 1 tested the ability of a multi-layered back-propagation network to recognise a set of upper and lower case letters presented in different fonts and sizes. The generalisation ability of the network was explored.

### 2.1 Network architecture and learning algorithm

**Fig. 1:**
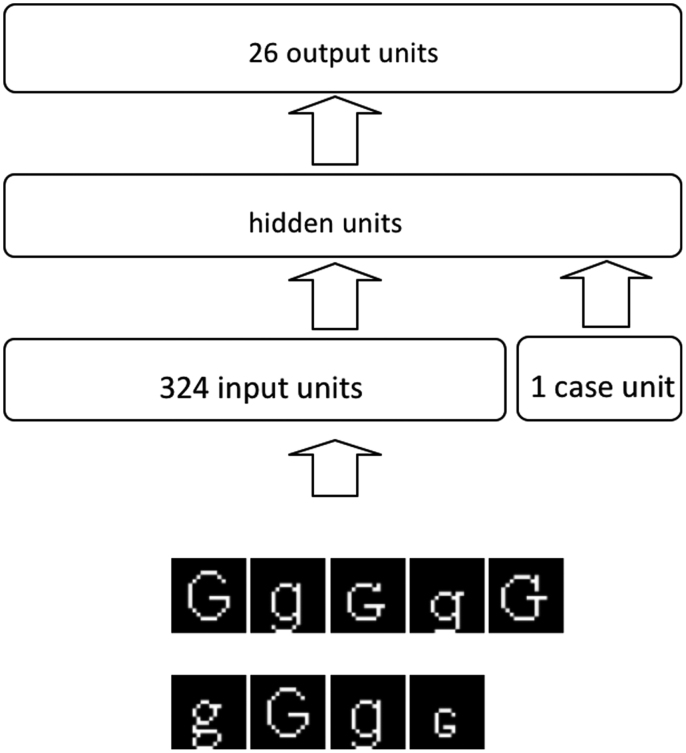
The architecture of the letter recognition network. The network's input is bitmap letter images in various different fonts and sizes.

Fig. 1 shows the architecture of the network. The network was a feedforward network and it consisted of three layers of units: 324 visual units, 50 hidden units, and 26 letter units. The number of hidden units was determined from pilot trials as the minimum number that provided perfect accuracy and optimal generalisation. There was an additional case unit to indicate whether the input is an upper case or lower case letter. This was used to mimic the situation that is ubiquitous in human confusability experiments where the subject is aware of what case letters they should expect because all letters are presented in either upper or lower case. The model is provided with this expectation via the case unit, but it should be noted that the model can accomplish recognition of all upper and lower case letters without this unit.

The case unit and each visual unit were connected to each unit at the hidden layer; similarly each hidden unit was connected to each unit at the letter output layer. The network was trained using the back-propagation algorithm with a learning rate of .1 and momentum of .9. A cross-entropy function was used to calculate the errors between the activation of the output unit and the target output. The activation of each unit was calculated by first determining the net input to that unit and then transforming the input using a logistic function to constrain the output activation between 0 and 1 according to the following equations:

(1) $$s_{i}=\sum_{j}w_{ji}a_{j}$$

(2) $$a_{i}=\frac{1}{1+e^{-s_{i}}}$$

In Eq. (1), *s*_*i*_ is the net input from all the projections *j* to the unit *i*, *a*_*j*_ is the activation of the units *j* and *w*_*ji*_ is the weight value between the units *j* and *i*. In Eq. (2), *a*_*i*_ is the activation of the unit *i*, which is the logistic transformation of its net input.

#### 2.1.1 Visual image representations

The network was trained with bitmap images of letters that were contained within an 18 by18 pixel grid. Each letter was presented in four different fonts: Arial; Courier New; Times New Roman and Helvetica, and five different sizes 8-, 9-, 10-, 11-, and 12-point. Both upper case and lower case letters were used. In addition letters in 5×7 Dot Matrix fonts were added to the training examples to allow for an exact replication of the stimuli from Gilmore et al.'s study (1979). Hence, there were in total 1033 letter images in the training corpus. The letters were represented in white against a black background. All the letters were positioned as they usually appear in text and were centred. Some lower case letters, for example, g, p, q and y, are placed below the line along which the text is organised. The line was set as the third row from the bottom on the 18 by 18 pixel grid. All the letter images were created by using the Matlab programming software. It is worth noting that these pixel based input representations probably do not represent a biologically realistic simulation of the input to the human visual system provided by the retina, which would have to capture sensitivity to colour and spatial frequency. However, the simplified visual representations used here do reflect the nature of the input to the retina itself as printed letters are usually constructed from very fine pixels. In any case we do not consider it is likely that the exact choice of visual input representations would affect the general conclusions of the paper.

#### 2.1.2 Frequency effect

In order to simulate human letter recognition realistically it is important to account for the effect of letter frequency. In Dewey's (1970) letter frequency count, the most frequent letter, E, accounts for 12.68% of all letters in English, while the least frequent letter, Z, accounts for .06% of all letters. In keeping with previous modelling works (Plaut et al., 1996; Seidenberg & McClelland, 1989; Welbourne & Lambon Ralph, 2007) a logarithmic function was employed to compress the effective frequency range. The compressed frequency is the logarithm of the actual frequency of a particular letter plus a constant *k*.

(3) LogFrequency(LF)=log(frequency+k)

*k* was chosen as the value of 1.057 to make the frequency compression ratio about 8:1. The range of the logarithmic frequencies was from .0165 to .1335. The compressed frequency values were used to scale error derivatives for the computation of weight updates.

#### 2.1.3 Training procedures

The network was trained using 95% of the letter images and the remaining randomly selected examples were used as the validation data set. This random removal was subject to the constraint that for any letter at least one example had to exist in each size. For each letter in the training set the input units were set and held to the value corresponding to the pixel of the letter image that they represented. Activation was propagated through the network and the output of each output unit was compared with its target value. Error was calculated using cross entropy and this error was used to calculate weight changes according to the standard back-propagation algorithm. The network was trained using full batches, which meant the weight changes were accumulated over all letter images and only updated when the full set had been completed. To avoid the possibility that the simulation result could be the consequence of one particular set of initial weights 10 networks were trained with 10 different random initial weights.

#### 2.1.4 Testing procedures

The performance of the network was tested on the training corpus every 100 epochs during the whole training period. The letter unit with the highest activation in the output layer was taken as the network response. The generalisation ability of the network was tested by using the validation data set, which had been withheld during training.

### 2.2 Results

**Fig. 2:**
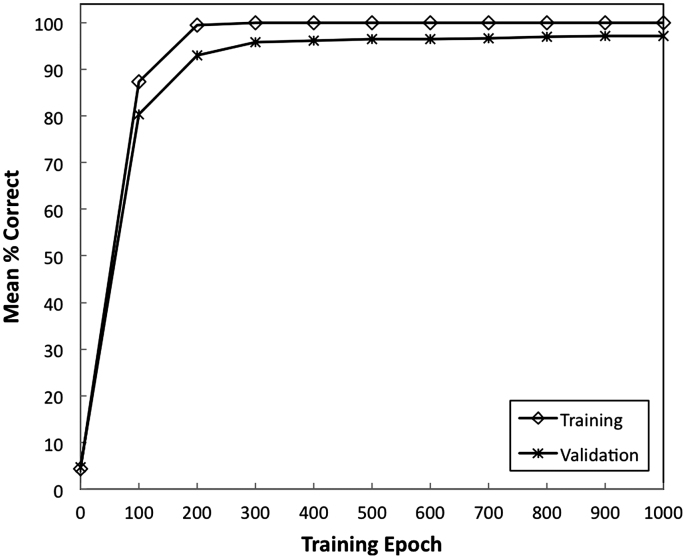
The average performance of the network on the training set and validation set as a function of training epoch.

Fig. 2 shows the average performance of the network as a function of the training epoch. The performance of the network followed a typical asymptotic learning trajectory with most of the learning occurring in the first 300 epochs. By epoch 500 the performance had reached asymptote and at this point the generalisation performance of the network was at 96.42%. The results showed the model can recognise all the letters in the training corpus and also achieve good performance on letters never seen before.

## 3 Simulation 2

A key objective for the current study is to examine how well the network could reproduce human confusability data and to explore the representational structure that underpins this; a good match between the model and human data would suggest that the model had extracted a similar set of features to support recognition and these features should be detectable in the structure of the hidden representations. In all the human studies letter confusion values are measured in a degraded viewing condition that gives approximately 50% letter identification accuracy. To simulate this in the model we added Gaussian blur to the input images mimicking the degraded viewing conditions of the human experiments.

### 3.1 Method

#### 3.1.1 Training procedures

The network was retrained on all the letter images including both the training and validation sets. All other training procedures are the same as in Simulation 1. The reason for training the network on all letters was to ensure that when it came to analysing the internal representations there would be no spurious effects arising from the fact that some letters were missing from the training set. To ensure that the network was still able to generalise an additional test was performed where the network was exposed to letters in fonts it had previously not seen. The network was tested on five different fonts Arial Unicode MS, Courier, Bookman, Verdana and Lucia Console with five different sizes 8-, 9-, 10-, 11- and 12-point.The network was able to recognise an average of 90.9% of these correctly indicating that this new method of training had not compromised the generalisation performance.

#### 3.1.2 Generating letter confusability matrices

**Fig. 3:**
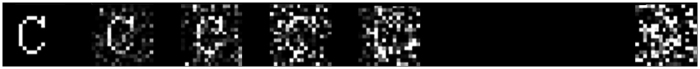
A example of letter C degraded with different amounts of noise.

**Fig. 4:**
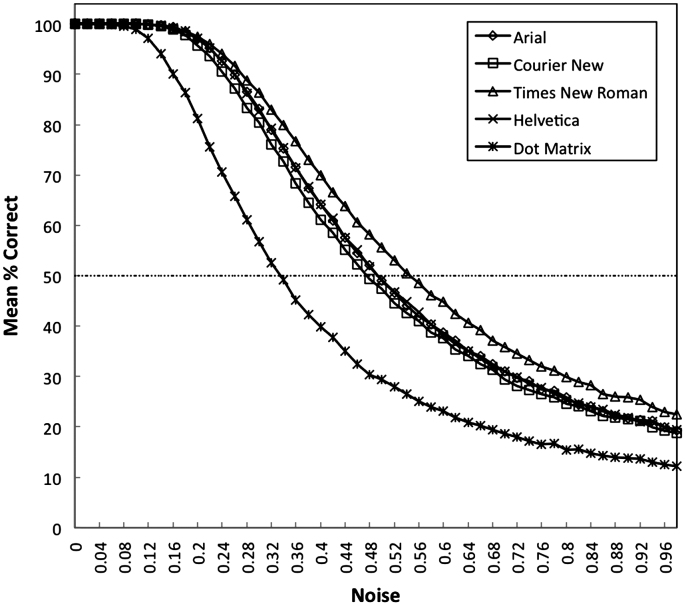
The performance of the network with Gaussian noise added to the input tested on five different fonts.

To generate the letter confusability matrices from the model, the first important procedure was to determine how much Gaussian noise should be added into the letter images to mimic a distorted viewing condition. As most letter confusion values in human data were measured by using upper case letters, the test stimuli were upper case letters including four different font sets of 10-point upper case letter images (Arial, Courier New, Times New Roman and Helvetica), and one set of 5×7 upper case letters in Dot Matrix font. Gaussian noise with standard deviation ranging from 0 to 1 in steps of .02 was added to each pixel in the image. An example of letter C degraded with different amounts of noise is shown in Fig. 3. Each noise condition was tested for 100 trials. The appropriate noise level to test confusability was selected separately for each of the five fonts to be the point where the performance of the network had decreased to 50% correct (see Fig. 4).

#### 3.1.3 Testing procedures for letter confusability matrices

After the appropriate distortion levels for each font had been determined, each of 26 upper case letters in each font was tested 150 times. A letter confusability matrix was computed by measuring the proportion of times each output unit was given as a response for each presented image letter. To assess the similarity between computer and human generated matrices, correlation coefficients were computed for the main-diagonal values and symmetrised off-diagonal values of the matrices (Loomis, 1982; Courrieu et al., 2004). The main-diagonal values of the confusability matrix represent the accuracy performance of the network on 26 letters. The more interesting data comes from the symmetrised off-diagonal values, which are the average confusability values of any two letters pairs. For example, the confusion value between letter E and letter F is the average of the confusability value for letter E recognised as letter F and the confusability value for letter F recognised as letter E. Four representative letter confusability studies were used as a measure of human letter confusability; details of these studies are summarised in Table 1.

### 3.2 Results

Tables 2A and 2B show the correlations between main-diagonal values and off-diagonal values of letters from all simulations and four previously reported human experiments. To help the interpretations of the results, Cohen's (1988, 1992) guidelines were applied in which the effect size of the correlation value smaller than .24 is considered small and the value greater than .36 is considered large, while any correlation value between these two boundaries indicates a medium effect. As shown in Table 2A, the simulations with Courier New font had significant main diagonal correlations with Townsend's (1971) and Loomis' (1982) data. There were also significant correlations between the simulation with Dot Matrix font and all four experiments except Van der Heijden et al. (1984) in which the highest one was with Gilmore et al.'s (1979) data. All of these correlations would be considered large according to Cohen's classification. The correlations between main-diagonal values of letters from all other simulations and experimental data were generally small. The letters in other fonts used in the network are different from those used in most letter confusability experiments (see Table 1). Only the 5×7 Dot Matrix font letters created are exactly the same as those used in Gilmore et al.'s (1979) experiment. It appears that the differences between fonts tend to reduce the correlations between recognition accuracy data. This is supported by the fact that the correlations between the human accuracy data in different fonts also varied from small to large values. Correlations between different fonts in the simulations also ranged widely. The large correlation between the Arial and Helvetica fonts reflects the fact that many of the letters are highly similar to each other. For the off-diagonal correlations (representing letter confusability in Table 2B) all the correlations were significant (and large by Cohen's classification); again the strongest correlation was found when the simulation used the same font letter as the human data (i.e., with the Dot Matrix font). These results suggest that confusability is highly dependent on font types. Unless the fonts are identical it is very difficult to reproduce the exact accuracy levels, and while the likely confusions are reasonably well predicted by the model using a different font, the best correspondence between human and simulation data is achieved when the fonts are an exact match.

In addition to consideration of the correlation data, it is also instructive to compare the confusions of individual letter pairs made by the model with the human data. We calculated the top four of human confusions for each letter to see whether it also appeared in the top four of the simulation confusions. Table 3 shows the result of this comparison where for each letter the first row shows the confusion data from Gilmore et al.'s (1979) study and the second row shows the data generated from the simulation with Dot matrix fonts as input. Those letter pairs that did not appear in the human confusions are marked in bold. Overall the match rate of the top four confusions made by human observers and the model was 60.58%, which is consistent with the correlation result. We also performed the same analyses to compare the two sets of human confusion data (i.e., Gilmore et al., 1979 vs. Loomis, 1982) which had the highest correlation confusions as shown in Table 2B. The analysis revealed that two sets of confusion data agreed 64.42% of the time in the top four confusions, which was close to Gilmore et al.'s data versus the simulation data. These results together with the correlation results suggest that the model made letter confusions, which were similar to those that can be observed in human subjects.

### 3.3 Analyses of the internal representations of letters in the model

The results heretofore demonstrate that the model can deal with the problem of size and shape invariance, generalise to previously unseen letters and produce human-like confusability patterns. This section is concerned with trying to understand the underlying representations that support these abilities and identifying what features are used for recognition in the model. Four analyses were conducted: (1) a principal components analysis was used to check that the representations of different sizes and cases of the same letter were similar; (2) hierarchical clustering analysis was used to explore the internal similarity structure of different letter representations; (3) the relationship between the confusability data and the internal representations was explored; (4) factor analysis was used to try and identify whether the model had extracted a set of visual feature detectors and, if so, which features were important in letter recognition.

#### 3.3.1 Similarity of same letter representations

**Fig. 5:**
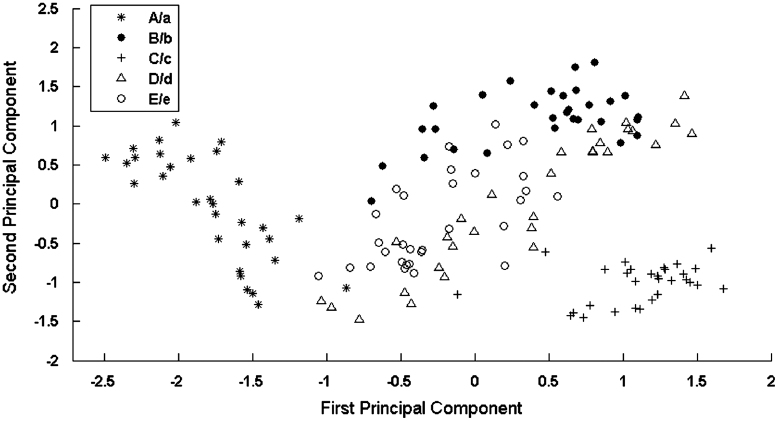
The relationship between the representations of five letter clusters [A/a, B/b, C/c, D/d ,E/e] with 41 transformations showing the first two principal components.

One method for assessing the representations of hidden units is to examine the similarity between the activations of all hidden units for all letters. The representations for a particular letter in different transformations should be very similar (i.e., their spatial distances should be very close to each other). To explore this visually principal component analysis was applied to identify the first two principal components of the multidimensional vector formed from the activations of the hidden units. Fig. 5 shows these components for five letter clusters [A/a, B/b, C/c, D/d, E/e] with all possible transformations including 8-point, 9-point, 10-point, 11-point and 12-point upper case and lower case letters. Each letter was seen in 41 distinct transformations, and it is clear that the representations form letter specific clusters, indicating that the internal representations of a particular letter in different transformations are similar. It is also worth noting that cluster A and cluster C are more distinctive from other clusters because they may be more dissimilar to others. While clusters B, E and D appear to have some overlaps indicating these letter clusters are more similar to each other. However, it is important to bear in mind that only two principal components were used to draw this figure, and the separation between letter clusters may look much clearer in a higher dimensional space.

#### 3.3.2 Similarity structure across letters

**Fig. 6:**
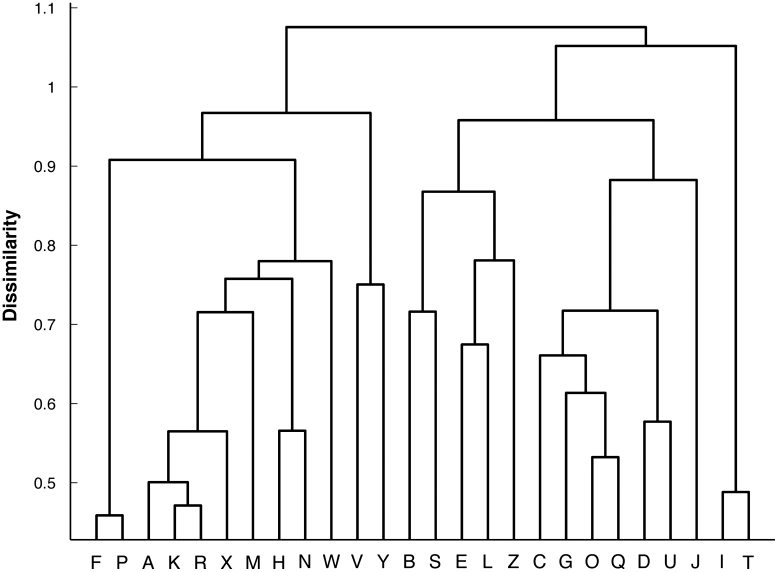
Hierarchical clustering plot of the letter similarity generated from the activations of the hidden units in the network with all upper case letters as input.

The internal similarity between representations for different letters was explored by using a hierarchical clustering technique. An agglomerative method was used to combine two clusters into a new cluster based on the average similarity of letters between two clusters. Fig. 6 shows the hierarchical clustering plot of letter similarity generated from the network using all the upper case letters. The most similar letter pair was the letter P and letter F. The letter pairs K and R, O and Q, and I and T were also very similar. It also can be observed that those letters which had unique angles or line terminations (e.g., M, H, N, W) were grouped together as were letters with a round shape (e.g., C, G, O, Q).

#### 3.3.3 Prediction of the letter confusability matrices

We have already shown that when the input is noisy the network can produce confusability matrices that have a significant correlation with human data. It would therefore be interesting to examine whether there are relationships between the internal representations generated from intact input and the confusability matrices generated from noisy input. If the similarity of the internal representations in the model can predict human confusability data then it suggests that this data is useful in helping us understand the internally extracted features that support letter recognition. The internal representations for each 10-point, upper case letter in each font were computed separately. The inter-letter inverse Euclidean distances from the internal representations were calculated for each letter pair. To examine whether the internal representations can predict the human confusion data, the correlations between the pairwise inter-letter inverse Euclidean distances and the symmetrised human confusability matrices were calculated (Table 4A). In addition, correlations were also computed with the confusion data from the network itself (Table 4B). All correlations with the human confusion data were significant. Correlations were higher when using the same font as the human experiment (i.e., the Dot Matrix font); consistent with the previous result confirming that confusability data is heavily influenced by font type. Confusability matrices are widely thought to be a useful tool to identify letter features (Bouma, 1971; Gilmore et al., 1979; Loomis, 1982; Townsend, 1971; Van der Heijden et al., 1984) and this result confirms that there is indeed a strong relationship between the internal representations of letters and the types of letter confusions that are produced.

#### 3.3.4 Key Features for letter recognition from internal representations

The results so far strongly suggest that the network has developed a feature based recognition system and that these features may be close to those used by human observers since they produce similar confusability matrices. Here we attempt to identify exactly what features of letters the network has learned to detect by using a rotated factor analysis applied to the activation values of the hidden units. Both upper and lower case letter data were entered into the analysis. Eight components were extracted, and the component matrix was rotated by using the orthogonal (varimax) method to allow for a better interpretation. The total variance explained by the eight components was 86%. The rotated component loading matrix and the total additional variance explained by each component are shown in Table 5. To interpret the data, we considered all values above .5 using two levels of significance: (1) loadings with values greater than .6 were marked in bold as highly important; (2) loadings with values between .5 and .6 underlined as important. Our interpretations of the eight components are listed below:

• The first component loaded positively on most letters with round curve shaped feature, for example, G, O, Q, U and u.
• The second component showed very high loadings on the letters such as A, K, R, X, a, h, k and n, which may be interpreted as the presence of an n-shaped feature on the bottom half of letters.
• The third component had very high loadings on the letters, which had a vertical central line feature such as I, T, i, l and t. It also loaded positively on Z, suggesting that the network may be misinterpreting the diagonal in Z as a central vertical line.
• The fourth component had high loadings on the letters B, F, P, b, p and r. The common feature among these letters seemed to be an inverted L shape or simply the lower case r-shape. Letters h and n, which also have this shape loaded highly on this factor as well.
• The fifth component loaded on to V, Y, v and y suggesting the detection of a V-shaped feature.
• The sixth component loaded on to E, Z, c, e and z, which seems to suggest the detection of a c-like feature.
• The seventh component loaded heavily on j, suggesting the detection of the hook-shaped feature. The component also loaded on s, g, S and J.
• The last component had a high loading on m and also loaded positively on W (loadings on M and w were also both above .4). This might suggest it responded to repeated vertical strokes.

These eight visual features contrast with the 16 features used by the IA model. The 16 IA features were used to construct letters in a specific font. They were all straight line segments including six vertical lines, six horizontal lines and four oblique lines with different orientations. Hence, the letters were composed of the combinations of those line segments without using any circular shapes. By contrast, the eight crucial features identified by the present model have some simple circular and angular shapes as well as the combinations of line segments in addition to direct line segments. These features have emerged from a purely statistical process of exposure to different letters in different fonts and transformations.

It should be noted that the use of the factor analysis is heavily dependent on the properties of the factor matrix and the rotation of the matrix, which could influence the interpretation of the features. However, the key point here is not the exact interpretation, but the fact that the network does extract statistically significant letter features which support recognition across a range of fonts, sizes and cases and can account for a very high percentage of variance in the hidden unit activations.

## 4 Simulation 3

According to the letter confusability account, the abnormally strong length effects observed in PA patients are driven by letter confusability (Arguin et al., 2002; Fiset et al., 2005; Fiset, Gosselin et al., 2006), suggesting patients are sensitive to the visual processing difficulty of letters.

This interpretation contrasts with the more generally held assumption that sequential letter processing leads to an emergence of strong length effects in PA patients (Warrington & Shallice, 1980). One aspect of the debate between these two accounts focuses on whether summed letter confusability or simple length best account for the slow reading of PA patients. These two variables carry theoretically different meanings, although they are highly correlated statistically. To explore the issue of whether word length or visual difficulty is a better factor for predicting PA patients' RTs, Simulation three calculated the summed error score for each letter and used it as a variable in a series of multiple regression analyses predicting PA patients' RTs for word reading. The error score generated from the simulation is a direct and accurate measure of the visual difficulty of recognising a particular letter and so allows us to further explore the question of whether PA patients' poor reading stems from a visual processing problem or an inability to engage parallel processing mechanisms. Of course visual difficulty and word length will be very highly correlated, as long words are intrinsically more difficult visually than short words. However our prediction would be that models that include a measure based directly on visual difficulty should predict a small but significant additional portion of the variance in patients RTs.

### 4.1 Patient data

Human naming latency data were taken from a case-series study on PA patients (Roberts Woollams, Kim, Beeson, Rapcsak, & Lambon Ralph, in press). In one of their experiments, 22 PA patients were tested on Weekes's (1997) monosyllabic word and nonword list with item lengths ranging from 3 to 6 letters. The stimuli consisted of 68 high frequency words, 68 low frequency words and 68 nonwords matched for number of phonemes, initial phoneme, orthographic neighbourhood size, number of enemies and friends, summed bigram frequency and average grapheme frequency to exclude potentially contaminating effects. All items were presented in lower case Courier New font.

**Fig. 7:**
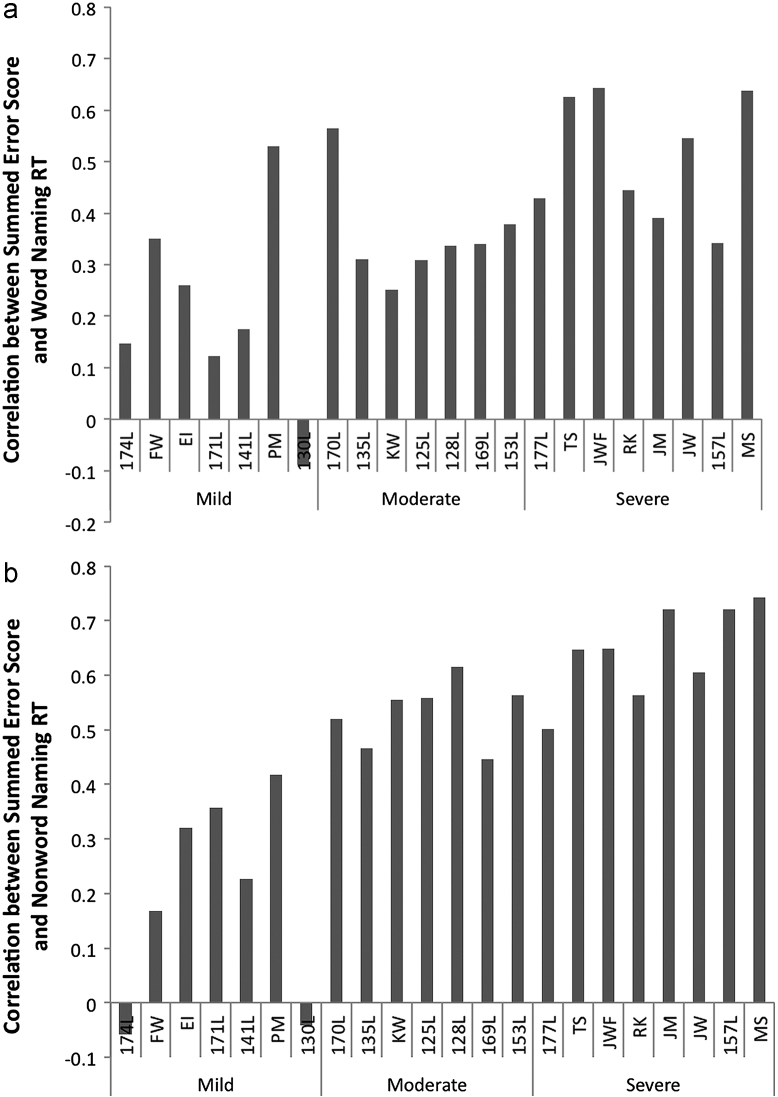
(A) Correlations between summed error score and individual patients' word naming RTs ordered by severity. (B) Correlations between summed error score and individual patients' nonword naming RTs ordered by severity.

This patient data encompasses a wide range of severity among the patients as indexed by overall reading speed. To account for the variance related to these severity differences the patients were categorised into three groups of severity based on their reading speed. Eight patients with RTs above 3000 ms were in the severe group, and others were equally divided into the mild and moderate groups with seven patients for each. RTs for these two groups ranged from 590 ms to 1144 ms and from 1650 ms to 2972 ms respectively. To examine the relationship between individual patients' data and summed error score, summed error score of all letters in a word was generated from the simulation when the input was noisy. The same font type (i.e., Courier New) as the stimuli in the human experiment was used. The correlation between patients' data and summed error score was computed. Fig. 7A shows the correlation between each patient's word naming RT and summed error score, arranged by severity. The result for nonword naming is shown in Fig. 7B. As can be seen, there is a general pattern that the correlation increases with severity, although it is not perfect and seems to be more consistent for the nonwords than the words. At the group level the correlations were clearly higher for the severe group than for the moderate group, and for the moderate group than for the mild group. These results suggest that there is an interaction between severity group and summed error score.

### 4.2 Multiple regression analyses on reaction time

Multiple regression analyses were used to examine: (1) whether models including summed error score would predict more of the variance in PA patients' RTs than models that only include word length; (2) whether there is an interaction between summed error score and severity group. To perform multiple regression analyses, any reaction time outside three standard deviations (SD) from the mean was filtered out. In addition to word length, PA patients are also sensitive to word frequency (Behrmann, Plaut et al., 1998; Johnson & Rayner, 2007; Montant & Behrmann, 2001) and orthographic neighbourhood size (Arguin et al., 2002; Fiset, Arguin et al., 2006; Montant & Behrmann, 2001). Both variables were also included into the multiple regression analyses. Word frequency was based on the CELEX frequency norm (Baayen, Piepenbrock, & Gulikers, 1995) and the logarithmic transform of frequency (Ln_CE_Freq) was used in the regression model to maximise the amount of variance that could be accounted for by this variable. Obviously the frequency factor was considered only for the regression on word naming latency and not for the nonword analysis. Orthographic neighbourhood size (OrthN) was taken from a database of the English Lexicon Project (Balota et al., 2007). Severity group was labelled numerically before being fed into the regression. To examine whether there was an interaction between severity group and summed error score, an interaction variable was also included in the regression. Similarly, another interaction predictor variable of severity group and word length was also entered into the model. Table 6A shows the inter-correlations between all the predictor variables and the simple correlations between patients' word naming RTs and the predictor variables were also included. As we had expected, word length was highly correlated with summed error score and orthographic neighbourhood size.

All the variables were simultaneously entered into the regression model to investigate which of these variables uniquely predicted a significant proportion of the variance. The result is shown in Table 7.

The regression model produced an *R*^2^ value of .472 (adjusted *R*^2^=.470), *p*<.001. Both log CELEX frequency and orthographic neighbourhood size made a significant contribution to account for the variance of RT. More importantly, severity group, summed error score and the interaction variable of summed error score by severity group, had significant unique effects, whilst word length was excluded from the regression model. For nonword naming, the inter-correlations between all the predictor variables and the simple correlations between patients' nonword naming RT and the predictor variables can be found in Table 6B. The regression result is also shown in Table 7. The *R*^2^ associated with the nonword analysis was .490 (adjusted *R*^2^=.487). Similar to the word analysis, severity group and summed error score were selected by the regression model and contributed significantly to the prediction of RTs, although the interaction of summed error by severity score was not significant. Word length was again excluded from the regression model and orthographic neighbourhood size also did not contribute significantly.

Several studies have shown that PA patients are also sensitive to other lexical variables, for instance, age-of-acquisition and imageability (Behrmann, Plaut et al., 1998; Behrmann et al., 2001; Cushman & Johnson, 2011; Fiset, Arguin et al., 2006). Unfortunately not all of the items used for analysis have ratings on these measures. However to see whether there was any evidence that an inclusion of these variables might affect the results of the regression on word naming latency, we performed an additional regression which included these two variables using values taken from the norms reported by Bird, Franklin, and Howard (2001), and Gilhooly and Logie (1980). The results showed that age-of-acquisition was a significant predictor while imageability was excluded from the regression model, as shown in Table 7. The significance of other variables was not changed and the variance accounted for was marginally increased to 47.4%.

Overall for both words and nonwords, the regression results suggest that summed error score may be a better predictor than word length for PA patients' reaction times when reading both words and nonwords. In addition, the results confirmed our previous observations that the effect of summed error score was modulated by severity, suggesting the severe patients are more sensitive to visual processing difficulty of words than are the milder patients. However, the high level of multicollinearity between word length and SES indicates that further investigation is required to confirm this.

### 4.3 Additional analyses to explore the influence of multicollinearity

The results of Section 4.2 have shown the summed error score was a stronger predictor than word length for PA patients' naming latencies. However, this interpretation of predictor variables requires further exploration because summed error score is highly correlated with word length, which will cause problems related to multicollinearity. Further collinearity diagnostic analyses showed that the eigenvalue of the last component was .002 and its condition index was 48.32. This component also had high variance proportions for WL and SES coefficients, which were .96 and .95 respectively. According to Myers and Well (2003), this would indicate the presence of a serious multicollinearity problem. To explore this issue, we performed three further analyses: (1) we ran separate regression models either with WL or SES as predictors and compared the total variance explained. (2) We regressed out all influence of WL from SES and used the residuals as an additional predictor in the WL model (we also conducted the reverse analysis regressing out all influence of SES from WL). (3) We conducted a factorial analysis where we constructed two subsets of stimuli consisting of four or five letters. In one set SES was matched across word lengths whereas the other set was not constrained by SES. If SES is the true driver of the length effect than we expected that there would be an interaction such that the word length effect would be abolished in the set that was matched for SES.

#### 4.3.1 Comparison of explained variance

One way to compare the predictive performance of summed error score and word length is to compare the amount of variance which can be accounted for by two regression models with either summed error score or word length coupled with all the other lexical variables^1^ used in the previous regression model. Both regression models then can be tested to see whether the *R*^2^ change is significant from the full model in Table 7. Thus, separate regression analyses were conducted for both word and nonword reading. The results showed that the model using SES as a predictor could account for slightly more variance than the model with word length as a predictor (*R*^2^=.472 versus .469), as reported in Table 8. This was also the case for the nonword reading where the model with SES predicted a slightly higher portion of variance than did the model with word length (*R*^2^=.489 versus .486). Importantly, when comparing with the full model (including all variables as predictors) the *R*^2^ change was significant for the length-based model while the change was not significant for the SES-based model. This suggests that the length-based model is a significantly worse predictor of word reading latency than the SES-based model. Similar results were obtained for nonwords, also shown in Table 8. Overall, these results confirmed the previous regression data and the regression analyses conducted thus far all show a consistent pattern. Although the difference in *R*^2^ values is small (.3% in both cases), this small difference is exactly what we would predict if we assume that the RTs of patients are primary driven by visual processing difficulty, which will inevitably be largely, but not completely, determined by word length.

#### 4.3.2 Analysis of the predictive ability of residual SES and WL scores

Another test of the reliability of the difference in predictive ability between SES and WL is to investigate whether a significant portion of variance can be explained by the residual of summed error score after the length factor is partialled out. If there is a unique portion of variance of reaction times which can be accounted for by the SES residual, this would suggest that summed error score contains useful information on visual difficulty, which cannot be obtained by using simple word length. To test this we regressed summed error score by word length to remove any covariance between them. Thus, there was no correlation between the SES residual and word length. The residual was then used as an additional predictor in the length-based regression model. As shown in Table 9, the regression results showed all variables accounted for a significant portion of variance for both word and nonword reading except that orthographic neighbourhood size was not a significant predictor in the nonword regression. Most importantly, the SES residual variables had significant unique effects in both word and nonword regression analyses. This conclusion was supported by the opposite test; using the residual obtained from regressing word length by summed error score in the SES-based models. In this case, the WL residual variables did not explain a significant portion of the variance in the word and nonword regression analyses. Comparison of the standardized betas for the residuals in the word and nonword cases suggests that the effect of the SES residuals was between 2.5 and 3.3 times greater than the effect of the WL residuals.

#### 4.3.3 Factorial approach

Another way to directly test the effect of summed error score generated by the network against word length is to test for an interaction pattern similar to that observed in Fiset et al.'s (2005) study. However, because of the limited number of words in Weekes's list, only four- and five-letter words could be used for test stimuli. Thus, two word lists were created and matched across lengths on Celex frequency and orthographic neighbourhood size. In the uncontrolled condition, words of different lengths were matched on the average error score of their constituent letters so that summed error score increased linearly with word length. While in the controlled condition, words were matched on their summed error score. There were 20 items in the uncontrolled condition and 14 items in the controlled condition. T tests comparing lexical variables of the words across different lengths showed no significant differences in frequency and orthographic neighbourhood size (all *p*s>.05).

**Fig. 8:**
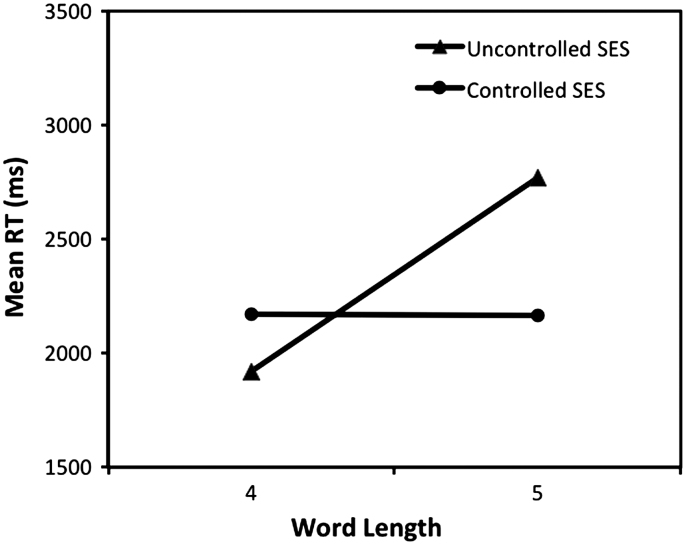
Mean reaction times for all patients with different matching conditions of summed error score.

A 2×2 ANOVA was performed on patients' average RTs, in which word length and condition (controlled versus uncontrolled) were used as between-item factors. The main effect of word length was significant, *F*(1, 30)=5.89, *p*=.021, while the main effect of condition was not, *F*(1, 30)=1.05, *p*=.313. Importantly, there was a significant interaction between word length and condition, *F*(1, 30)=6.08, *p*=.020, as shown in Fig. 8. Although the range of word lengths is slightly smaller than the 5–7 letters used in Fiset et al.'s study, the result produced a similar interaction pattern to that seen in Fiset et al.'s (2005; Fig. 1) data, showing an absence of word-length effects when summed error score was matched across lengths, while the effects persisted where SES was matched on a per letter basis.

Taken together, despite the potential issues of multicollinearity, these additional results strongly suggest that visual processing difficulty, rather than simple word length, is the key factor driving response times in pure alexic patients.

## 5 General discussion

This study describes a neural network model that can recognise printed letters in a variety of sizes and fonts including some that it was not trained on. When presented with visually degraded stimuli the network generated a font dependant letter confusability matrix similar to that obtained from human observers viewing degraded stimuli in the same font. During training the network developed a set of feature detectors that supported recognition of letters in various sizes and fonts. The network was also able to provide a good account of impaired reading in PA patients. A regression model including summed error score taken from the network was able to predict the word naming latencies of a group of 22 PA patients with the model accounting for 47% of the variance. Overall the findings suggest that visual processing difficulty, as indexed by summed error score (SES), is the key factor that determines PA patients' reaction times. This factor is very closely related to simple word length, but the analyses conducted here suggest SES can account for more unique variance than WL. Crucially, controlling for SES eliminates the WL effect. Unlike the IA model (McClelland & Rumelhart, 1981) and its derivatives, the current network was able to learn to extract features as a consequence of exposure to the training examples. Thus the feature set used to support recognition becomes an emergent property of the network's experience. This means it is not limited to the recognition of a specific font type as there is always the possibility of learning new features that are required for additional fonts. The current network provides an important link between visual input and the development of letter features. The generalisation performance of the network was 96.42% correct on the validation set, indicating that the model was able to recognise letters never seen before. Further explorations of the network's generalisation showed that the network could also recognise letters in novel fonts.

A key finding from this study is that the network has the ability to simulate letter confusability data obtained from human subjects. Letter confusability is often used to try and identify which features are crucial for human letter recognition (Bouma, 1971; Gilmore et al., 1979; Loomis, 1982; Townsend, 1971; Van der Heijden et al., 1984). The comparison between simulations and experimental data provide a sense of the similarity between features extracted by the model and those used in human letter recognition tasks. When training on the same font letters as those used in the experiments, the network can account for the confusability data obtained from the human subjects. The importance of font type as a factor in determining the similarity between different confusability matrices is highlighted by the experimental data reported in Tables 2A and 2B, where studies using similar techniques and similar levels of degradation still produce considerable differences in the correlations between the identification accuracies and confusion values if the fonts are not similar. Another approach to measuring confusability is to use inverse discrimination times. Using this approach Courrieu et al. (2004) also obtained significant correlations with the existing confusability studies (Bouma, 1971; Jacobs et al. 1989) but the values were only at the moderate level. It is possible that the use of different techniques for degrading the stimuli complicated the outcomes (Courrieu et al., 2004). However, based on our findings another possible explanation for these discrepancies is the use of different font types. This explanation may be consistent with Pelli et al.'s (2006) finding that font types are particularly important to letter recognition because the visual complexities of the same letter in different fonts can be very different.

Data analyses of the internal representations of the network showed significant correlations between human confusion values and similarities in the internal representational structure. Factor analysis of this representational structure suggested that recognition was supported by the detection of eight visual features. These were simple shapes including: a round curve; an n-shape; a vertical line; an inverted L; a V-shape; a c-like feature; a hook-shape and repeated vertical strokes. Although Fiset et al. (2008, 2009) found that line termination is the most important feature for recognising both lower and upper case letters in Arial font, they also reported additional useful feature sets for recognising individual letters based on the classification images and these show some interesting similarities and differences with our own data. For instance, the round curve shaped feature (Table 5) shared by some upper case letters like C, O and U also can be observed in Fiset et al. (2009; Fig. 2). However, in our data the upper case letters G and Q also had high loadings on this component, while Fiset et al. (2009) reported the most important features were the horizontal bar and terminations for letter G and the terminations (i.e., the lower short bar) for Q. It is important to note that this does not necessarily mean that letters G and Q do not have a curve shaped feature according to Fiset's analysis. In fact the differences between the analyses may be largely due to the use of different feature identification techniques. The bubbles technique employed by Fiset et al. (2008, 2009) is particularly useful for revealing small unique features such as line terminations and intersections, which are the most salient features for individual letters. By contrast, the factor analysis conducted here is designed to identify a common set of features shared across letters. These two techniques therefore tend to address different aspects of features for letter recognition with Fiset et al. emphasising unique features while our data emphasises shared features.

Further evidence for the generality of the model comes from its ability to explain a considerable fraction of the variance in the reading times of PA patients. Using summed error score to directly measure visual processing difficulty, we demonstrated that summed error score was slightly better at predicting RT than word length, accounting for a significant additional .3% of the variance (based on 22 PA patients' reading data). These results held for both word naming and nonword naming. Although this difference is relatively small, it is theoretically important, because sensitivity to summed error score suggests that the primary deficit in these patients is related to visual processing rather than the adoption of a serial processing strategy. Summed error score and word length are very closely correlated so it is conceivable that this conclusion may not be valid because of the problems with multicollinearity. However, further analyses conducted in Section 4.3 confirmed that summed error score carries additional information over and above word length, which makes it better than word length alone for predicting PA patients' RTs. Crucially controlling for SES in a subset of the stimuli eliminates the word length effect. Therefore it is possible to conclude that visual processing difficulty is the key factor in explaining PA patients' RTs. In addition there was an interaction between summed error score and severity, indicating that severe patients are more sensitive to this factor. These findings provide support for the peripheral visual deficit account of pure alexia, which also explains why patients are sensitive to the visual similarity between letters (Fiset et al. 2005). Additionally, it is worth noting that patients' reading times were significantly affected by frequency, age-of-acquisition and orthographic neighbourhood size. These results are consistent with previous studies showing that some PA patients are sensitive to lexical factors including word frequency (Behrmann, Plaut et al., 1998; Montant & Behrmann, 2001), age-of-acquisition (Cushman & Johnson, 2011), and orthographic neighbourhood size (Arguin et al., 2002; Fiset, Arguin et al., 2006; Montant & Behrmann, 2001), suggesting that some top-down influences are still being activated by the visual stimuli. This might lead to stronger top-down effects in PA patients who need to compensate for the degraded bottom-up signals (Behrmann, Plaut et al., 1998; Johnson & Rayner, 2007; Montant & Behrmann, 2001).

The main limitation of this model is that it can only recognise isolated letters and it is unable to address the role of context in recognition. Further work might extend the present model to become a full word reading model and train the network with feedback connections from the word layer back to the letter input layer. In the current model, the output layer could be considered as an area coding for abstract letter identities corresponding to a brain region within the left fusiform gyrus, also known as the visual word form area (VWFA). This area is thought by many (Cohen et al., 2000, 2004, 2002) to be specific to the processing of visual word forms and it is certainly true that damage to the VWFA itself or the connections to and from this area results in impaired letter recognition as seen in PA patients. However, an alternative account is that the left fusiform gyrus region is for generic object processing and is not limited to visual word form processing (Devlin, Jamison, Gonnerman, & Matthews, 2006; Price & Devlin, 2003). In the context of PA patients the conflicting interpretations of the precise function of VWFA might be partly due to exactly what source of behavioural data is used. Studies that suggest an exclusively word-form specific role for VWFA tend to assume that word recognition is impaired because of the length effect in RT, but that object recognition is intact because patients can pass standard object recognition tests that focus on accuracy rather than RT. The object recognition accuracy of PA patients is often within a normal range (Farah & Wallace, 1991; Starrfelt, Habekost, & Leff, 2009). However, several recent studies have found that PA patients show RT deficits in object recognition tasks (Behrmann, Nelson et al., 1998; Mycroft, Behrmann, & Kay., 2009) similar to their RT deficits in words; suggesting a general visual impairment rather than a purely orthographic problem. Further support for this view comes from a study by Woodhead et al. (2011), which shows that the left VWFA is preferentially tuned to high spatial frequencies, whereas the right analogue area is more sensitive to lower frequencies. This would suggest that the VWFA should be more sensitive to word stimuli, which carry more information in higher spatial frequencies than do faces or objects. However, damage to the area would still be expected to impair performance on all classes of visual stimuli, provided a sufficiently sensitive test is used.

The model presented here could be extended to do simple object recognition tasks as well as letter recognition to help with the examination of these two contrasting theories in the pure alexia literature.

In summary, the current model is able to develop its own representations to capture a set of visual features that support letter recognition of both learned and novel letters in a variety of sizes and fonts. It also shows sensitivity to letter confusability and network derived confusability matrices correlate well with human data provided that they are using the same font. We demonstrate that network generated summed error score (i.e., a measure of visual processing difficulty) is the key factor in determining the reaction times of this group of PA patients.

## Figures and Tables

**Table 1:** Summary of four letter confusability experiments.

**References**	**Procedure**	**Presentation**	**Font/size**	**Case**
Townsend (1971)	The stimulus exposure sequence was white pre-stimulus with fixation point, stimulus with a random letter, and post-stimulus field with fixation point. Each letter was black against a white background. The luminance for all stimuli was 5.6 fL. The subject specific stimulus durations were chosen to produce 50% correct performance	Tachistoscope	Typewriter font/8×11 in.	Upper case
Gilmore et al. (1979)	Each letter was green in colour against a dark background. The fixation point was presented for 1 s at the beginning of each trial, and the stimulus letter was briefly presented 1 s after the fixation point disappeared. Then, it was followed by a blank screen. The stimulus durations for subjects ranged from 10 to 70 ms to obtain the correct response rate of .5	Computer	5×7 Dot Matrix	Upper case
Loomis (1982)	The visual stimuli were the transparent characters subjected to optical low-pass spatial filtering prior to viewing. The visual stimuli were viewed with the right eye in a darkened room. The stimulus duration was 2 s. The correct recognition performance was about .6	Shutter	Degraded Helvetica Extra Light/5.7 mm height	Upper case
van der Heijden et al. (1984)	Each letter was white against a dark background and subtended a visual angle of .32°×.48°. The fixation point was displayed until the subjects clearly saw the fixation point. After that, the stimulus letter was briefly presented and the subjects had to respond with a letter name within 500 ms. The stimulus durations for subjects ranged from 3 to 18 ms to obtain the correct response rate of .5	Computer	Sans Serif Roman	Upper case

**Table 2A:** Correlations for diagonal values between letter confusability matrices from simulations and experimental data.

	Gilmore et al. (1979)	Loomis (1982)	van der Heijden et al. (1984)	AR simu.	CN simu.	TNR simu.	HEL simu.	DM simu.
Townsend (1971)	.212	.387	.477^⁎^	.339	.537^⁎⁎^	.278	.347	.464^⁎^
Gilmore et al. (1979)		.684^⁎⁎^	.503^⁎⁎^	.004	.269	−.029	.045	**.566**^⁎⁎^
Loomis (1982)			.538^⁎⁎^	.164	.503^⁎⁎^	.197	.212	.479^⁎^
van der Heijden et al. (1984)				−.056	.363	.279	−.037	.346

AR simu.					.528^⁎⁎^	.360	.991^⁎⁎^	.321
CN simu.						.548^⁎⁎^	.538^⁎⁎^	.582^⁎⁎^
TNR simu.							.347	.248
HEL simu.								.330

Note: ^⁎⁎^Significant (*p*<.01); ^⁎^Significant (*p*<.05). AR: Arial; CN: Courier New; TNR: Times New Roman; HEL: Helvetica; DM: Dot Matrix; Simu.: Simulation.

**Table 2B:** Correlations for symmetrised off-diagonal values between letter confusability matrices from simulations and experimental data.

	Gilmore et al. (1979)	Loomis (1982)	van der Heijden et al. (1984)	AR simu.	CN simu.	TNR simu.	HEL simu.	DM simu.
Townsend (1971)	.552^⁎⁎^	.612^⁎⁎^	.680^⁎⁎^	.587^⁎⁎^	.469^⁎⁎^	.482^⁎⁎^	.572^⁎⁎^	.484^⁎⁎^
Gilmore et al. (1979)		.747^⁎⁎^	.692^⁎⁎^	.560^⁎⁎^	.556^⁎⁎^	.381^⁎⁎^	.538^⁎⁎^	**.670**^⁎⁎^
Loomis (1982)			.680^⁎⁎^	.584^⁎⁎^	.522^⁎⁎^	.465^⁎⁎^	.556^⁎⁎^	.503^⁎⁎^
van der Heijden et al. (1984)				.598^⁎⁎^	.557^⁎⁎^	.484^⁎⁎^	.582^⁎⁎^	.617^⁎⁎^

AR simu.					.657^⁎⁎^	.649^⁎⁎^	.988^⁎⁎^	.653^⁎⁎^
CN simu.						.688^⁎⁎^	.653^⁎⁎^	.649^⁎⁎^
TNR simu.							.647^⁎⁎^	.478^⁎⁎^
HEL simu.								.651^⁎⁎^

Note: ^⁎⁎^Significant (*p*<.01); ^⁎^Significant (*p*<.05). AR: Arial; CN: Courier New; TNR: Times New Roman; HEL: Helvetica; DM: Dot Matrix; Simu.: Simulation.

**Table 3:** Comparison of the top four confusion values between human letter confusions and confusions made by the network with the Dot Matrix font as input.

Rank	1	2	3	4
A	A-R (.133)	A-H (.081)	A-N (.042)	A-M (.040)
	A-N (.091)	A-H (.093)	A-R (.058)	**A-P (.049)**
B	B-G (.067)	B-D (.059)	B-S (.058)	B-R (.051)
	**B-E (.090)**	B-S (.084)	**B-P (.067)**	B-R (.046)
C	C-G (.121)	C-E (.049)	C-O (.040)	C-N (.032)
	**C-L (.010)**	C-O (.091)	C-G (.082)	C-E (.075)
D	D-O (.107)	D-B (.059)	D-G (.053)	D-Q (.040)
	**D-L (.060)**	**D-U (.059)**	D-Q (.057)	D-O (.048)
E	E-G (.052)	E-B (.050)	E-C (.049)	E-F (.041)
\	E-B (.090)	E-C (.075)	E-F (.061)	**E-L (.052)**
F	F-P (.159)	F-E (.041)	F-R (.040)	F-K (.028)
	F-P (.156)	F-K (.085)	F-R (.068)	F-E (.061)
G	G-C (.121)	G-O (.113)	G-Q (.112)	G-S (.081)
	G-Q (.126)	G-C (.082)	G-O (.068)	**G-J (.058)**
H	H-M (.227)	H-W (.221)	H-N (.205)	H-A (.080)
	H-M (.124)	H-N (.092)	H-A (.090)	H-W (.063)
I	I-T (.063)	I-Z (.020)	I-E (.015)	I-L (.013)
	I-T (.194)	**I-Y (.045)**	**I-K (.033)**	I-L (.033)
J	J-U (.024)	J-Z (.018)	J-I (.012)	J-G (.009)
	J-U (.078)	**J-Q (.06)**	J-G (.058)	**J-S (.050)**
K	K-X (.111)	K-R (.068)	K-E (.041)	K-H (.036)
	K-R (.087)	**K-F (.085)**	**K-W (.05)**	**K-M (.047)**
L	L-N (.045)	L-E (.033)	L-K (.020)	L-G (.013)
	**L-C (.105)**	**L-D (.060)**	L-E (.052)	**L-U (.037)**
M	M-H (.227)	M-N (.113)	M-W (.056)	M-A (.040)
	M-H (.124)	M-N (.111)	M-W (.070)	**M-X (.059)**
N	N-H (.205)	N-W (.116)	N-M (.113)	N-L (.045)
	N-W(.127)	N-M (.111)	N-H (.092)	**N-A (.091)**
O	O-Q (.142)	O-G (.113)	O-D (.111)	O-B (.047)
	**O-C (.091)**	O-Q (.069)	O-G (.068)	**O-U (.065)**
P	P-F (.159)	P-R (.076)	P-D (.036)	P-B (.030)
	P-F (.156)	P-R (.135)	P-B (.067)	**P-A (.049)**
Q	Q-O (.142)	Q-G (.112)	Q-D (.040)	Q-R (.033)
	Q-G (.126)	Q-O (.069)	**Q-J (.060)**	Q-D (.057)
R	R-A (.133)	R-P (.076)	R-K (.068)	R-B (.051)
	R-P (.135)	R-K (.087)	**R-F (.068)**	R-A (.058)
S	S-G (.081)	S-B (.058)	S-R (.032)	S-E (.027)
	S-B (.084)	**S-Z (.060)**	**S-J (.050)**	**S-A (.039)**
T	T-I (.063)	T-P (.027)	T-Y (.027)	T-F (.021)
	T-I (.194)	T-Y (.066)	T-F (.035)	**T-S (.023)**
U	U-V (.042)	U-O (.041)	U-W (.035)	U-Q (.032)
	**U-J (.078)**	U-O (.065)	**U-D (.059)**	U-Q (.045)
V	V-Y (.113)	V-U (.042)	V-B (.029)	V-D (.024)
	V-Y (.054)	**V-R (.013)**	**V-G (.012)**	**V-I (.011)**
W	W-H (.221)	W-N (.116)	W-M (.056)	W-U (.035)
	W-N (.127)	W-M (.070)	W-H (.063)	**W-X (.059)**
X	X-K (.111)	X-N (.043)	X-M (.040)	X-R (.037)
	X-M (.059)	**X-W (.059)**	X-K (.034)	X-N (.033)
Y	Y-V (.113)	Y-T (.027)	Y-P (.011)	Y-X (.011)
	Y-T (.066)	Y-V (.054)	**Y-M (.051)**	**Y-I (.045)**
Z	Z-E (.033)	Z-C (.029)	Z-X (.022)	Z-I (.020)
	**Z-S (.060)**	Z-E (.047)	**Z-Y (.028)**	**Z-R (.021)**

*Note*: in each block, the first row shows the data from Gilmore et al.'s (1979) study and the second row is the simulation data based on the Dot Matrix font. For those letter-pairs which do not appear in the top four confusions of human data are marked in bold.

**Table 4A:** Correlations for symmetrised off-diagonal values and inter-letter inverse Euclidean distances from the internal representations of each upper case letter in each font generated by the network.

	AR inver. Euclid. dist.	CN inver. Euclid. dist.	TNR inver. Euclid. dist.	HEL inver. Euclid. dist.	DM inver. Euclid. dist.
Townsend (1971)	.445^⁎⁎^	.505^⁎⁎^	.472^⁎⁎^	.445^⁎⁎^	.437^⁎⁎^
Gilmore et al. (1979)	.565^⁎⁎^	.510^⁎⁎^	.388^⁎⁎^	.565^⁎⁎^	**.708**^⁎⁎^
Loomis (1982)	.522^⁎⁎^	.515^⁎⁎^	.424^⁎⁎^	.522^⁎⁎^	.531^⁎⁎^
van der Heijden et al. (1984)	.550^⁎⁎^	.533^⁎⁎^	.474^⁎⁎^	.550^⁎⁎^	.556^⁎⁎^

Note: ^⁎⁎^Significant (*p*<.01); ^⁎^Significant (*p*<.05). AR: Arial; CN: Courier New; TNR: Times New Roman; HEL: Helvetica; DM: Dot Matrix; Inver. Euclid. Dist.: Inverse Euclidean Distance.

**Table 4B:** Correlations between the confusion values from the network and the inter-letter inverse Euclidean distances from the internal representations of each upper case letter in each font generated by the network.

Font type	Correlations
Arial	.717^⁎⁎^
Courier New	.762^⁎⁎^
Times New Roman	.650^⁎⁎^
Helvetica	.712^⁎⁎^
Dot Matrix	.847^⁎⁎^

Note: ^⁎⁎^Significant (*p*<.01); ^⁎^Significant (*p*<.05).

**Table 5:** Principal components analysis (with varimax rotation) loadings.

	Component							
	1	2	3	4	5	6	7	8
A	.258	.**811**	.046	.182	.184	.078	.069	.211
B	.494	.425	.066	.**625**	.110	.068	−.138	−.003
C	.**789**	−.154	.364	.029	−.092	.285	−.105	−.003
D	.**729**	.145	.003	.339	.059	−.226	−.284	−.037
E	.271	.525	.307	.247	.261	.516	−.233	−.062
F	−.233	.448	.184	.**688**	.263	.172	−.094	−.092
G	.**905**	.122	.074	.014	.003	−.002	.109	.205
H	.307	.**685**	−.252	.352	.245	−.136	−.177	.158
I	.080	−.094	.**957**	−.064	.061	−.045	.091	−.079
J	.598	−.086	.169	−.282	.118	.074	.463	−.415
K	.195	.**867**	.108	.152	.110	.100	.068	.068
L	.361	.269	.430	.221	−.026	.193	−.491	−.213
M	.164	.**610**	−.114	.002	.539	.013	−.224	.404
N	.417	.**669**	−.232	.264	.119	−.299	.034	.119
O	.**809**	−.114	.092	.066	−.300	.075	−.089	.328
P	−.112	.386	.031	.**777**	.384	−.065	−.029	−.021
Q	.**832**	.121	−.018	−.076	−.208	−.155	.137	.201
R	.136	.**832**	−.030	.280	.291	.059	.001	−.037
S	.558	.180	.296	.283	.125	.207	.435	.021
T	.028	.098	.**877**	.022	.206	.128	.086	.046
U	.**871**	.115	−.156	.088	.090	−.189	−.272	−.023
V	.056	.296	.057	.091	.**842**	−.063	−.132	−.112
W	.347	.504	.031	.428	.022	.011	.214	.526
X	−.099	.**824**	.049	.024	.274	.244	.261	.035
Y	−.138	.201	.302	−.069	.**778**	.175	.206	.104
Z	.145	.270	.514	−.283	.066	.**653**	.085	−.164

a	.594	.514	.031	−.024	.050	.344	.157	.104
b	.458	.291	.059	.**734**	−.057	.079	−.114	.108
c	.552	−.110	.312	.254	−.056	.**637**	−.059	.021
d	.**789**	.247	−.119	−.052	.054	.348	.105	−.077
e	.487	.320	.137	.316	.055	.578	−.055	.226
f	−.371	.113	.**646**	.447	.045	.390	−.068	.052
g	.598	.269	−.127	.091	.084	.122	.395	−.060
h	.234	.**683**	−.138	.594	.000	−.025	−.142	.009
i	−.065	−.049	.**932**	−.027	.138	.085	.001	.015
j	−.052	.222	.313	−.349	−.005	−.096	.**736**	−.167
k	−.058	.**823**	.158	.306	.205	.237	.053	.049
l	−.066	−.135	.**888**	.087	−.215	.164	.029	−.005
m	.215	.367	.158	−.137	.266	.098	−.177	.**721**
n	.485	.541	−.188	.531	−.177	.032	−.089	−.022
o	.**787**	−.034	.005	.361	−.271	.238	−.002	.228
p	.265	.179	−.038	.**802**	.014	−.149	.117	.051
q	.**752**	.344	−.132	.007	−.121	.102	.192	−.054
r	−.245	.170	.309	.**667**	.083	.481	−.159	.014
s	.359	.242	.262	.332	.031	.339	.512	.072
t	−.021	.142	.**768**	.129	.030	.384	.113	.241
u	.**894**	.105	−.193	.088	.047	.054	−.156	−.073
v	−.051	.259	−.009	.180	.**813**	.146	−.128	.031
w	.372	.461	.031	.387	−.253	−.014	.177	.498
x	−.174	.**675**	.089	.129	.324	.320	.385	.077
y	−.221	.190	.019	.096	.**792**	−.052	.313	.081
z	.113	.218	.398	−.111	.097	.**802**	.122	.062
% Of variance explained by each component	20.98	17.12	11.93	11.25	8.11	7.45	5.24	3.93

**Table 6A:** Correlations between predictor variables and word naming RT used in the regression model.

		WL	OrthN	SES	Group	WL_Group	SES_Group	LnCE_Freq
IV	WL	1						
	OrthN	−.754^⁎⁎^	1					
	SES	.963^⁎⁎^	−.761^⁎⁎^	1				
	Group	−.034	.031	−.030	1			
	WL_Group	.002	−.009	.005	−.013	1		
	SES_Group	.005	−.012	.007	−.011	.964^⁎⁎^	1	
	LnCE_Freq	−.079^⁎⁎^	.219^⁎⁎^	−.114^⁎⁎^	.027	0	−.001	1

DV	RT(s)	.197^⁎⁎^	−.193^⁎⁎^	.207^⁎⁎^	.604^⁎⁎^	.178^⁎⁎^	.186^⁎⁎^	−.144^⁎⁎^

Note: ^⁎⁎^Correlation is significant at the .01 level; ^⁎^Correlation is significant at the .05 level. WL: word length; OrthN: orthographic neighbourhood size; SES: summed error score. Group: severity group; SES_Group: the product of summed error score and group; WL_Group: the product of word length and group; Ln_CE_Freq: log CELEX frequency.

**Table 6B:** Correlations between predictor variables and nonword naming RT used in the regression model.

		WL	OrthN	SES	Group	WL_Group	SES_Group
IV	WL	1					
	OrthN	−.732^⁎⁎^	1				
	SES	.953^⁎⁎^	−.738^⁎⁎^	1			
	Group	−.047	.057	−.059	1		
	WL_Group	.001	−.020	.001	0	1	
	SES_Group	.001	−.017	.001	−.002	.952^⁎⁎^	1

DV	RT(s)	.193^⁎⁎^	−.132^⁎⁎^	.191^⁎⁎^	.629^⁎⁎^	.201^⁎⁎^	.200^⁎⁎^

Note: ^⁎⁎^Correlation is significant at the .01 level; ^⁎^Correlation is significant at the .05 level; WL: word length; OrthN: orthographic neighbourhood size; SES: summed error score; Group: severity group; SES_Group: the product of summed error score and group; WL_Group: the product of word length and group.

**Table 7:** Regression coefficients of all predictor variables.

Predictor	Word		Restricted word		Nonword	
	Beta	*P* value	Beta	*P* value	Beta	*P* value
WL	.049	.388	.051	.364	.056	.460
OrthN	−.049	.038^⁎^	−.045	.059	.012	.715
SES	.124	.029^⁎^	.118	.038^⁎^	.185	.016^⁎^
Group	.617	<.001^⁎⁎^	.617	<.001^⁎⁎^	.642	<.001^⁎⁎^
WL_Group	.012	.828	.013	.811	.093	.208
SES_Group	.179	.001^⁎⁎^	.177	.002^⁎⁎^	.113	.124
LnCE_Freq	−.131	<.001^⁎⁎^	−.122	<.001^⁎⁎^	–	–
AoA	–	–	.046	.003^⁎⁎^	–	–
IMG	–	–	−.018	.222	–	–

*R*^2^ (%)	47.2		47.4		49.0	

Note: ^⁎⁎^Correlation is significant at the .01 level; ^⁎^Correlation is significant at the .05 level. Beta coefficients presented were based on the standardized scores. WL: word length; OrthN: orthographic neighbourhood size; SES: summed error score; Group: severity group; SES_Group: the product of summed error score and group; WL_Group: the product of word length and group; Ln_CE_Freq: log CELEX frequency; AoA: age-of-acquisition; IMG: imageability.

**Table 8:** Comparison of regression coefficients and *R*^2^ change (from the full model) for separate Length-based and SES-based models.

	Word						Nonword					
	Length-based			SES-based			Length-based			SES-based		
	Beta	*P*		Beta		*P*	Beta		*P*	Beta	*P*	
WL	.162	<.001^⁎⁎^		–		–	.220		<.001^⁎⁎^	–	–	
OrthN	−.059	.013^⁎^		−.052		.026^⁎^	−.003		.925	.008	.810	
SES	–	–		.169		<.001^⁎⁎^	–		–	.235	<.001^⁎⁎^	
Group	.618	<.001^⁎⁎^		.617		<.001^⁎⁎^	.640		<.001^⁎⁎^	.643	<.001^⁎⁎^	
WL_Group	.185	<.001^⁎⁎^		–		–	.200		<.001^⁎⁎^	–	–	
SES_Group	–	–		.191		<.001^⁎⁎^	–		–	.202	<.001^⁎⁎^	
LnCE_Freq	−.134	<.001^⁎⁎^		−.129		<.001^⁎⁎^	–		–	–	–	

*R*^2^ (%)	46.9			47.2			48.6			48.9		

	*F* change		*P*	*F* change	*P*		*F* change	*P*		*F* change		*P*

*R*^2^ change (from the full model in Table 7)	7.36		.001^⁎⁎^	.39	.68		4.17	.02^⁎^		1.07		.34

Note: ^⁎⁎^Correlation is significant at the .01 level; ^⁎^Correlation is significant at the .05 level; Beta coefficients presented were based on the standardized scores. WL: word length; OrthN: orthographic neighbourhood size; SES: summed error score; Group: severity group; SES_Group: the product of summed error score and group; WL_Group: the product of word length and group; Ln_CE_Freq: log CELEX frequency.

**Table 9:** Regression coefficients of all predictor variables in the length-based model with the SES residual and the SES-based model with the WL residual in predicting PA patients' reaction times.

	Word				Nonword			
	Length-based		SES-based		Length-based		SES-based	
	Beta	*P*	Beta	*P*	Beta	*P*	Beta	*P*
WL	.169	<.001^⁎⁎^	–	–	.232	<.001^⁎⁎^	–	–
OrthN	−.050	.036^⁎^	−.049	.038^⁎^	.013	.703	.012	.732
SES	–	–	.172	<.001^⁎⁎^	–	–	.237	<.001^⁎⁎^
Group	.617	<.001^⁎⁎^	.617	<.001^⁎⁎^	.642	<.001^⁎⁎^	.643	<.001^⁎⁎^
WL_Group	.185	<.001^⁎⁎^	–	–	.201	<.001^⁎⁎^	–	-
SES_Group	–	–	.191	<.001^⁎⁎^	–	–	.202	<.001^⁎⁎^
LnCE_Freq	−.131	<.001^⁎⁎^	−.131	<.001^⁎⁎^	–	–	–	–
SES residual	.033	.033^⁎^	–	–	.056	.015^⁎^	–	–
WL residual	–	–	.013	.390	–	–	.017	.455

*R*^2^ (%)	47.0		47.2		48.9		48.9	

Note: ^⁎⁎^Correlation is significant at the .01 level; ^⁎^Correlation is significant at the .05 level; Beta coefficients presented were based on the standardized scores. WL: word length; OrthN: orthographic neighbourhood size; SES: summed error score; Group: severity group; SES_Group: the product of summed error score and group; WL_Group: the product of word length and group; Ln_CE_Freq: log CELEX frequency; SES Residual: residual of regressing SES by WL; WL Residual: residual of regressing WL by SES.

## Footnotes

1 Other lexical variables here include log Celex frequency, orthographic neighbourhood size, severity group and the interaction term. Age-of acquisition and imageability are not considered because, as mentioned, not all of the items used for analysis have ratings on these measures.
